# P208: Detection of an outbreak through routine surveillance and its control

**DOI:** 10.1186/2047-2994-2-S1-P208

**Published:** 2013-06-20

**Authors:** P Barman, S Sengupta, R Pande, M Puri

**Affiliations:** 1 Microbiology, BLK Super speciality hospital , New Delhi, India; 2 Critical Care Medicine, BLK Super speciality hospital , New Delhi, India

## Introduction

Ventilator associated pneumoniae (VAP) is a major burden of healthcare associated infections (HAI) and leading cause of death of mortality and morbidity in critical care patients. NHSN 2010 reported a VAP pooled mean of 1.2 per 1000 ventilator days. The INICC study reported higher VAP rates in developing countries. VAP rates of 10.4 were reported from India. Surveillance has always been an effective tool for reduction of VAP worldwide. The importance of surveillance is not only to detect infection risk, process and outcome but also as a tool for recognizing outbreaks.

## Objectives

To detect, investigate and control an outbreak of pneumoniae in ventilated patients in the medical ICU at a tertiary care hospital.

## Methods

Prospective surveillance for HAI is routinely carried out following NHSN/ CDC definitions. Analysis and feedback of this data is presented to the clinicians by the infection control team (ICT) every quarter.

## Results

The VAP rates from January 2011 to July 2011 were between 2.5 to 5.52 per 1000 ventilator days. A marginal increase (7 per 1000 ventilator days) was seen during August and September 2011.A sharp rise followed in the month of December and January 2012 (16.08 to22.84 per 1000 ventilator days respectively). The ICT alerted the critical care team. An investigation was launched and an outbreak of Acinetobacter pneumoniae in ventilated patients was detected. A case definition was established and extent of outbreak assessed through line listing of cases, laboratory investigations and outcome. A fish bone analysis was done of the various processes involved. It resulted that the ambu bags which were used for patients harbored the Acinetobacter inspite of disinfection. The disinfection protocol was modified and training was imparted to staff involved in disinfection of the ambu bags. A checklist was introduced for monitoring all ventilated patients and strict implementation of VAP bundles was initiated. Surveillance following the above steps over the next three months showed a significant decrease in VAP rates in the intensive care unit (3.4 to 6.4 per 1000 ventilator days).

## Conclusion

It has been proved that routine prospective surveillance is an excellent tool to combat HAIs and proves effective in detection of outbreaks which otherwise would have gone unnoticed.

## Disclosure of interest

None declared

